# Successful treatment with repeated dexamethasone implant injections for recurrent macular edema after acute retinal necrosis

**DOI:** 10.1186/s12348-022-00310-5

**Published:** 2022-10-21

**Authors:** Ragnhild Øvstebø Sørland, Anne Kjersti Erichsen, Thora Elisabet Jonsdottir, Marius Nordberg Bromnes, Peter Mæhre Lauritzen, Jon Roger Eidet

**Affiliations:** 1 Department of Ophthalmology, Oslo University Hospital, Oslo, Norway; 2 Department of Radiology, Oslo University Hospital, Oslo, Norway

**Keywords:** Acute retinal necrosis, Cystoid macular edema, Dexamethasone implant, Valacyclovir

## Abstract

**Background:**

The treatment of recurrent cystoid macular edema associated with acute retinal necrosis is challenging due to the concern that treatment with intravitreal steroids may reactivate the retinitis.

**Case report:**

An immunocompetent patient diagnosed with acute retinal necrosis was treated with oral valacyclovir and intravitreal injections of foscarnet. Giant tears in her retina necessitated a vitrectomy with silicone oil. She developed cystoid macular edema after the removal of the silicone oil. The edema responded to high-dose prednisolone but recurred when the dose was tapered to 20 mg daily. Under close surveillance and increased antiviral medication, she was treated with a dexamethasone implant with complete resolution of the edema. Unfortunately, the edema recurred, and the treatment had to be repeated. Over 18 months, she received five dexamethasone implants without recurrence of the viral retinitis.

**Conclusions:**

This case shows successful treatment of recurring cystoid macular edema following acute retinal necrosis with repeated intravitreal dexamethasone implants in a patient receiving valacyclovir maintenance treatment.

## Introduction

Acute retinal necrosis (ARN) is a rare and potentially blinding disease [9]. Cystoid macular edema (CME) is one of many complications causing poor visual outcomes in ARN patients [11], and the most common cause of permanent visual loss among uveitis patients [2, 7]. The treatment of CME in uveitis patients can be challenging because the edema often recurs [1]. Corticosteroids are the mainstay in treating non-infectious uveitic CME [1]. However, in the case of infectious uveitis, caution should be taken. There is no consensus about the treatment of CME secondary to ARN, nor is there any consensus on the dosage of antiviral medications when patients are treated with intravitreal steroids.

## Case report

**Fig. 1:**
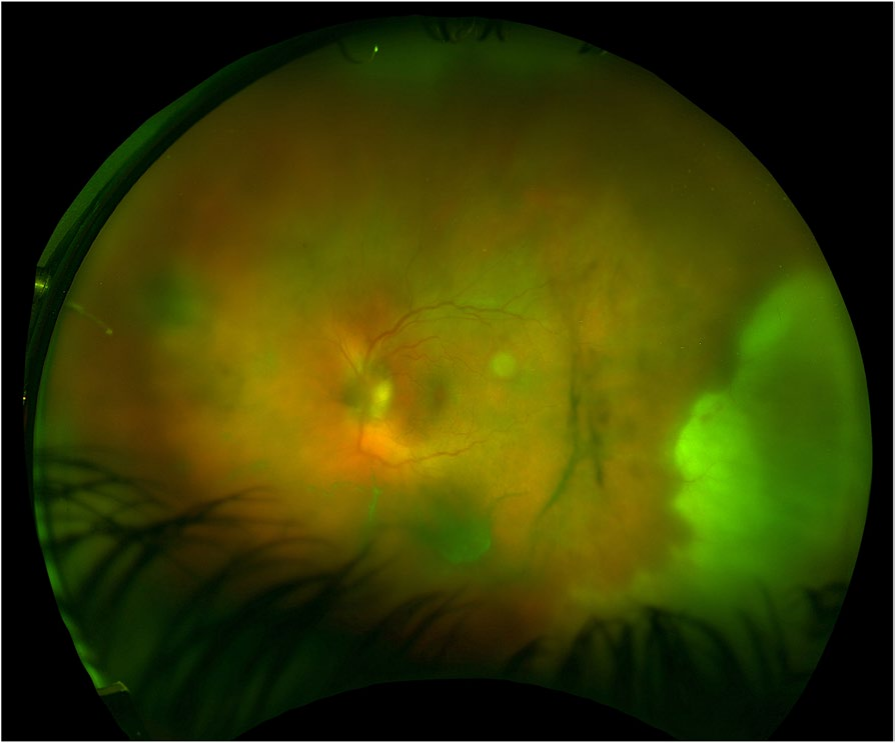
Optomap fundus photography shows inflammatory optic disc edema and peripheral confluent areas of a necrotic retina

An immunocompetent woman in her early twenties was referred to The Department of Ophthalmology in Oslo with a painful left eye and reduced vision. Her best-corrected visual acuity (BCVA) was 20/100 (Snellen equivalent), and a slit-lamp examination revealed 3+ cells in the anterior chamber and vitritis. Fundus examination showed inflammatory optic disc edema and peripheral confluent areas of a necrotic retina (Fig. 1). There was no pathology in her right eye. Vitreous sampling for polymerase chain reaction (PCR) analysis confirmed the presence of DNA from the herpes simplex virus type 2 (HSV-2). She was diagnosed with acute retinal necrosis, and oral induction therapy with valacyclovir 2 g four times daily was initiated. She received four intravitreal injections of 2.4 mg foscarnet in 0.1 ml during the first nine days of admission. Due to the optic disc edema, she was started on oral prednisolone at 40 mg daily, which was gradually tapered and discontinued after three months.

Giant tears in the necrotic retina complicated the initial diagnostic vitrectomy. This necessitated a second vitrectomy two weeks later with silicone oil and prophylactic laser retinopexy and cryopexy. Her BCVA after surgery was 20/30, and optical coherence tomography (OCT) showed no sign of macular edema. After removal of the silicone oil five months after the second vitrectomy, she developed increased inflammation in the anterior chamber and CME. The CME responded to oral prednisolone at 60 mg daily but recurred when tapering the dose to 20 mg daily. Even though a minor CME remained, prednisolone was discontinued to avoid oral steroid-related adverse effects.

Within the first year, valacyclovir was slowly tapered to a maintenance dose of 500 mg three times daily. Eighteen months after the first admission she had developed a mature cataract that required surgery. While still on valacyclovir 500 mg three times daily, prednisolone 60 mg daily was reintroduced pre-operatively to control inflammation. Postoperatively there was no CME, and her BCVA was 20/30. The CME recurred when prednisolone was tapered to 20 mg daily, and her BCVA declined to 20/100, both due to CME and the development of posterior capsular opacification (PCO). She then received one intravitreal injection of 0.05 ml aflibercept with no apparent effect on the CME.

**Fig. 2:**
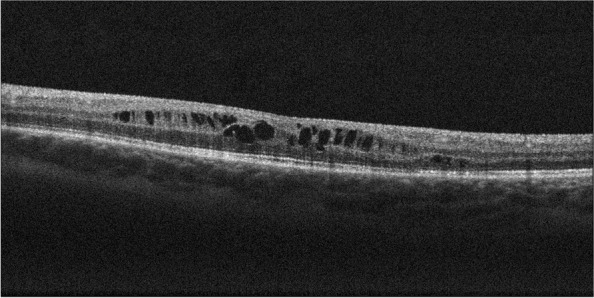
Optical coherence tomography shows cystoid macular edema

**Fig. 3:**
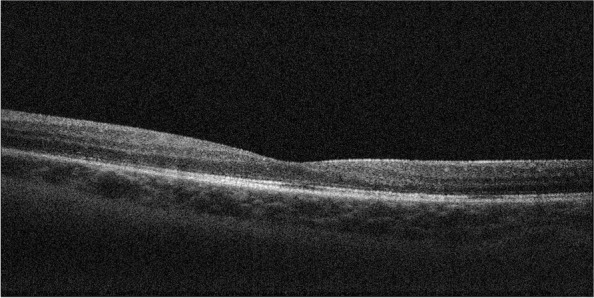
Optical coherence tomography shows complete resolution of the cystoid macular edema four weeks following dexamethasone implant injection

As the treatment of the CME necessitated unacceptably high doses of oral prednisolone, and the edema was unresponsive to injection with aflibercept, intravitreal steroid injections were initiated. The valacyclovir maintenance dose was doubled to 1 g three times daily before injecting intravitreal triamcinolone 4 mg in 0.1 ml. One week after the injection, there was an improvement in the CME. However, the BCVA was still 20/100 due to PCO, and a YAG capsulotomy was performed. Two months later, the CME was worsening (Fig. 2), and while still on valacyclovir 1 g three times daily, an intravitreal 700 ug dexamethasone implant was injected. Four weeks later, there was a complete resolution of the CME (Fig. 3), and her BCVA had improved to 20/50. Two months after the first dexamethasone implant, valacyclovir maintenance therapy was reduced to 500 mg three times daily.

The patient received five dexamethasone implants over 18 months while maintaining her BCVA level at 20/40. The injection interval was between 12 and 18 weeks. She was monitored closely with regular fundus exams and intraocular pressure measurements. There was no sign of reactivation of retinitis.

## Discussion

Acute retinal necrosis is an infectious retinitis caused by members of the herpes virus family [6]. In the absence of antiviral medication, corticosteroids should be used cautiously as this may promote viral replication [8, 10]. Although oral corticosteroids are routinely used to treat acute inflammation and optic disc edema in ARN patients [11], there is concern that steroids may reactivate the retinitis when injected intravitreally.

Macular edema will cause permanent damage to the anatomic structures in the macula and irreversible visual loss if left untreated. Our patient had a BCVA level of 20/30 when her macular edema was treated with high-dose prednisolone. Unfortunately, long-term use of systemic corticosteroids may have serious adverse effects [1], so an alternative treatment modality was needed. Vascular endothelial growth factor inhibitors have been tried in non-infectious uveitic macular edema with variable results [3, 5]. Ortega-Evangelio et al. [5] reported a case of CME secondary to ARN successfully treated with intravitreal aflibercept. However, aflibercept did not improve the CME in our patient. Majumder et al. 2016 [4] reported two cases of CME in ARN patients, in one of which PCR confirmed the varicella-zoster virus. The CME in each case was successfully treated with two dexamethasone implant injections three months apart while using valacyclovir 1 g three times daily. In contrast, our patient had ARN due to HSV-2, and her CME was successfully treated with five dexamethasone implant injections over 18 months. In addition, the retinitis did not reactivate in our patient even though she used a lower valacyclovir maintenance dosage than reported by Majumder et al.

In this patient, repeated intravitreal dexamethasone implants with concomitant valacyclovir maintenance treatment proved to be an effective long-term treatment for CME secondary to ARN.

## Data Availability

Not applicable.

## Abbreviations

ARN: Acute retinal necrosis
CME: Cystoid macular edema
BCVA: Best corrected visual acuity
PCR: Polymerase chain reaction
HSV-2: Herpes simplex virus type 2
OCT: Optical coherence tomography
PCO: Posterior capsular opacification
